# Benefit assessment in Germany: implications for price discounts

**DOI:** 10.1186/s13561-016-0109-3

**Published:** 2016-08-02

**Authors:** Ulrike Theidel, J-Matthias Graf von der Schulenburg

**Affiliations:** 1 Xcenda GmbH, Lange Laube 31, 30159 Hannover, Germany; 2 Center for Health Economics Research Hannover (CHERH), Leibniz Universität Hannover, Otto-Brenner-Straße 1, 30159 Hannover, Germany

**Keywords:** Health policy, Pharmaceuticals, Market access, Rebate, Negotiation, New substance

## Abstract

**Background:**

The AMNOG regulation, introduced in 2011 in Germany, changed the game for new drugs. Now, the industry is required to submit a dossier to the GBA (the central decision body in the German sickness fund system) to show additional benefit. After granting the magnitude of the additional benefit by the GBA, the manufacturer is entitled to negotiate the reimbursement price with the GKV-SV (National Association of Statutory Health Insurance Funds). The reimbursement price is defined as a discount on the drug price at launch. As the price or discount negotiations between the manufacturers and the GKV-SV takes place behind closed doors, the factors influencing the results of the negotiation are not known.

**Objectives:**

The aim of this evaluation is to identify factors influencing the results of the AMNOG price negotiation process.

**Methods:**

The analysis was based on a dataset containing detailed information on all assessments until the end of 2015. A descriptive analysis was followed by an econometric analysis of various potential factors (benefit rating, size of target population, deviating from appropriate comparative therapy and incorporation of HRQoL-data).

**Results:**

Until December 2015, manufacturers and the GKV-SV finalized 96 negotiations in 193 therapeutic areas, based on assessment conducted by the GBA. The GBA has granted an additional benefit to 100/193 drug innovations. Negotiated discount was significantly higher for those drugs without additional benefit (*p* = 0.030) and non-orphan drugs (*p* = 0.015). Smaller population size, no deviation from recommended appropriate comparative therapy and the incorporation of HRQoL-data were associated with a lower discount on the price at launch. However, neither a uni- nor the multivariate linear regression showed enough power to predict the final discount.

**Conclusions:**

Although the AMNOG regulation implemented binding and strict rules for the benefit assessment itself, the outcome of the discount negotiations are still unpredictable. Obviously, negotiation tactics, the current political situation and soft factors seem to play a more influential role for the outcome of the negotiations than the five hard and known factors analyzed in this study. Further research is needed to evaluate additional factors.

## Background

The Act on the Reform of the Market for Medical Products (*Arzneimittelmarkt-Neuordnungsgesetz,* AMNOG) became effective in 2011. Upon market registration, pharmaceutical companies are obliged by law to submit a dossier to the Federal Joint Committee (*Gemeinsamer Bundesausschuss*, GBA) in order to prove a patient-relevant medical benefit in mortality, morbidity, and health-related quality of life (HRQoL). The GBA is the decision-making body of the joint self-governing board of stakeholders in healthcare (physicians, dentists, hospitals and sickness funds) in the German Statutory Health Insurance (*Gesetzliche Krankenversicherung*, GKV). The methodological basis of the underlying assessment and the uncertainties regarding outcomes and study results is in accordance with the principles of evidence-based medicine. The Institute for Quality and Efficiency in Health Care (*Institut für Qualität und Wirtschaftlichkeit im Gesundheitswesen,* IQWiG) publishes its own methods on assessments in a specific method paper including key elements on how assessments are to be carried out [1].

A ruling on additional benefit for a specific compound by the GBA (appraisal) is based on the overall assessment of the evidence presented in the dossier for effects on mortality, morbidity, and HRQoL. Besides a rating based on self-assessment by the manufacturer when the dossier is submitted, an assessment from the IQWiG and/or the GBA is vital for the final benefit rating [2]. Sometimes a manufacturer decides not to submit a dossier and accepts a rating of no additional benefit from the beginning.

As mentioned, the acknowledgement of a patient-relevant medical benefit by the GBA is crucial. An independent assessment of the evidence by the IQWiG supports the decision by the GBA. As an institution, designated by law, IQWiG supports the GBA with providing independent, evidence-based expert reports. IQWiG submits the results of their assessment to the GBA with a recommendation regarding the additional patient-relevant benefit of the investigated drug. Overall, the GBA decision is based on the manufacturers’ dossier and submitted data, the IQWiG evaluation, as well as the results of the public hearing as part of the commenting procedure.

The following categories define the magnitude of additional benefit: (1) major, (2) considerable, (3) minor, (4) non-quantifiable, (5) no additional benefit, or (6) less benefit. The number and characteristics of studies provided, the certainty of results, and the observed effects determine the level or quality of evidence (‘proof’, ‘indication’ or ‘hint’) [3]. The GBA decides about the additional benefit of a new compound compared to the appropriate comparative therapy (ACT) usually named within the scientific advice. The final decision on the ACT is taken by the GBA when deciding on the added benefit ultimately.

Price negotiations begin after a final decision on the (additional) medical benefit, announced by the GBA. The negotiation procedure contains mainly the negotiation of a discount on the published list price in addition to already existing mandatory discounts in accordance with §130a of the 5^th^ Social Law Book. Representatives of the manufacturer negotiate the price with representatives of the National Association of Statutory Health Insurance Funds (*Spitzenverband Bund der Krankenkassen*, GKV-SV). The negotiations have to be finalized within 6 months after market access. In case that no final agreement is achieved on price discount after an additional 6 months, an arbitration will be initiated. During a 3-month timeframe, an official arbitration board will set the final price decision. Companies are free to set the price for the first 12 months after market launch, the negotiated (or arbitrated) discount becomes effective immediately after this first year [4].

Until now, no published analyses have been conducted addressing factors that might influence the magnitude of a discount. Therefore, the aim of this evaluation was to identify potential factors influencing final negotiated discounts.

## Methods

### Conceptual framework and analytic strategy

Based on the “hunches of experts”, we have identified several factors, which might influence the final discount. Factors influencing the negotiated discount on the price of a new drug are many and we had to be selective in this study to ensure that the analysis is significant. Five potential influencing factors were scoped in our analysis, which are assumed as most relevant:

1. Rating (as the result of the benefit assessment)
2. Indication class
3. Orphan drug status (also reflected by size of target population)
4. ACT (depending on number and annual costs)
5. HRQoL data (when incorporated in the assessment).

### Data sources

The underlying dataset contains all dossiers with benefit ratings published and negotiated until December 31^st^, 2015. Excluding those admitted to a reference price group or choosing opt-out, all benefit assessments were analyzed with respect to these factors and their impact on negotiated discounts. Besides these factors, other potential differentiators were scoped.

For this analysis, we evaluated the discount per therapeutic area assessed. It has to be noted, that one new drug might have been subject to one or more assessments, for instance after a label extension, requiring a reassessment and/or the need to split up a single process in one or more therapeutic areas (e.g. the usual number of therapeutic areas in diabetes is five).

The basis for calculation of discounts were price information published by the official Germany drug price list (“Lauertaxe”). Prices and discounts for the time at start of negotiations and end of negotiations were gathered and used for the analysis. Label extensions might change the price and negotiated discounts. In that case, we considered the latest discount update.

### Statistical analysis

We analyzed the data in a two-step approach with IBM SPSS Statistics 23. First, to describe this comprehensive data sample with respect to the assumed potential influence factors, we report quantitative variables, e.g. mean, standard deviation, minimum and maximum.

Second, based on our description, we derived some hypotheses on what influences the price. Hereby we conducted a linear regression model to analyze the impact of these factors on the final discount. The dependent variable in the model was the price change (discount) of the new therapy.

The independent variables in the regression model were as mentioned above and we decided to

1. build a univariate regression for each independent variable,
2. build a multivariate regression model including all variables with *p* < 0.05.

## Results

### Descriptive analysis

Manufacturers and GKV-SV finalized price negotiations for 193 assessed therapeutic areas within 96 assessments until December 31^st^, 2015. The GBA granted an additional benefit for 100/193. Of these, one was major, 29 were considerable, 48 minor, and 22 non-quantifiable additional benefit ratings. One rating was with additional harm potential (=less benefit). Manufacturers presented no dossier for five assessments.

#### Rating

The overall mean discount was 21.96 % [0–78.02; SD 13.14] on the initially freely set price at launch. The mean discount for “no additional benefit” (*n* = 93) of 24.09 % [0–78.02; SD 14.19] was significantly higher than discount for an “additional benefit” (*n* = 100) 19.98 % [0–58.73; SD 11.81] (*p* = 0.030). One assessment resulted in a “major additional benefit” (13.64 %) and one in “additional harm potential” (9.00 %). Overall, two indication classes showed significant results for the mean discount compared to the others: “Diseases of the Respiratory System” and “Diseases of the Nervous System”. They present the minimum and the maximum mean discount when comparing discounts per indication class (Tables 1 and 2).

**Table 1:** Discounts by benefit categories and indication class

Additional benefit: overall					
Indication class by ICD-10	*n*	Mean	Min	Max	SD
Certain infectious and parasitic diseases	51	22.01	0.00	42.54	12.00
Diseases of the blood and blood-forming organs	2	24.50	23.15	25.85	1.91
Diseases of the circulatory system	14	25.25	4.65	58.73	16.10
Diseases of the digestive system	6	27.42	14.87	31.02	6.52
Diseases of the eye and adnexa	7	19.46	3.06	78.02	26.57
Diseases of the genitourinary system	2	19.80	19.80	19.80	0.00
Diseases of the musculoskeletal system and connective tissue	1	39.44	39.44	39.44	–
Diseases of the nervous system	9	37.84 *	24.32	67.30	14.40
Diseases of the respiratory system	9	12.93 *	2.01	25.74	11.24
Diseases of the skin and subcutaneous tissue	1	42.75	42.75	42.75	–
Endocrine, nutritional and metabolic diseases	24	19.86	1.00	48.60	12.43
Mental and behavioral disorders	2	25.68	17.36	34.00	11.77
Neoplasms	57	20.01	0.00	53.71	11.16
Other	8	20.73	13.64	28.55	6.43
Overall ^a^	193	21.96	0.00	78.02	13.14
Size of target population ^a^ (upper level)	193	122808	6	3253000	409065
Additional benefit: overall with additional benefit					
Indication class by ICD-10	*n*	Mean	Min	Max	SD
Certain infectious and parasitic diseases	29	20.16	0.00	42.54	12.57
Diseases of the circulatory system	8	27.32	4.65	58.73	17.77
Diseases of the digestive system	2	20.21	14.87	25.54	7.54
Diseases of the eye and adnexa	1	15.43	15.43	15.43	–
Diseases of the genitourinary system	2	19.80	19.80	19.80	0.00
Diseases of the musculoskeletal system and connective tissue	1	39.44	39.44	39.44	–
Diseases of the nervous system	2	40.69 *	29.71	51.67	15.53
Diseases of the respiratory system	3	4.99 *	2.01	10.96	5.17
Endocrine, nutritional and metabolic diseases	11	14.11	1.00	25.31	7.60
Other	4	16.36	13.64	24.50	5.43
Neoplasms	37	20.09	4.74	46.44	9.66
Overall	100	19.98	0.00	58.73	11.81
Size of target population (upper level)	100	80418	6	2542810	302323

*Abbreviations*: *Min* minimum, *Max* maximum, *SD* standard deviation

All discounts presented in %

*p < 0.05 compared to mean discount of the other indications

^a^Including the assessment with major benefit

**Table 2:** Discounts by benefit categories and indication class

Additional benefit: considerable					
Indication class by ICD-10	*n*	Mean	Min	Max	SD
Certain infectious and parasitic diseases	11	23.80	10.07	42.54	13.96
Diseases of the circulatory system	1	19.35	19.35	19.35	–
Diseases of the eye and adnexa	1	15.43	15.43	15.43	–
Diseases of the musculoskeletal system and connective tissue	1	39.44	39.44	39.44	–
Endocrine, nutritional and metabolic diseases	1	5.05	5.05	5.05	–
Neoplasms	14	18.44	6.35	35.19	8.69
Overall	29	20.66	5.05	42.54	11.55
Size of target population (upper level)	29	18172	143	221000	40788
Additional benefit: minor					
Indication class by ICD-10	*n*	Mean	Min	Max	SD
Certain infectious and parasitic diseases	12	18.48	0.00	42.54	13.78
Diseases of the circulatory system	6	29.97	4.65	58.73	20.21
Diseases of the digestive system	1	25.54	25.54	25.54	–
Diseases of the genitourinary system	2	19.80	19.80	19.80	0.00
Diseases of the nervous system	2	40.69	29.71	51.67	15.53
Diseases of the respiratory system	2	2.01 *	2.01	2.01	0.00
Endocrine, nutritional and metabolic diseases	10	15.02	1.00	25.31	7.36
Neoplasms	12	23.16	4.74	46.44	12.83
Other	1	24.50	24.50	24.50	–
Overall	48	20.93	0.00	58.73	20.16
Size of target population (upper level)	48	153723	6	2542810	425313
Additional benefit: non-quantifiable					
Indication class by ICD-10	*n*	Mean	Min	Max	SD
Certain infectious and parasitic diseases	6	16.86	10.07	21.25	5.33
Diseases of the circulatory system	1	19.35	19.35	19.35	–
Diseases of the digestive system	1	14.87	14.87	14.87	–
Diseases of the respiratory system	1	10.96	10.96	10.96	–
Neoplasms	11	18.84	9.00	29.78	6.29
Other	2	13.64	13.64	13.64	0.00
Overall	22	17.31 *	9.00	29.778	55.32
Size of target population (upper level)	22	6081	25	34000	9994
No additional benefit					
Indication class by ICD-10	*n*	Mean	Min	Max	SD
Certain infectious and parasitic diseases	22	24.45	3.16	42.54	11.01
Diseases of the digestive system	4	31.02	31.02	31.02	0.00
Diseases of the blood and blood-forming organs	2	24.50	23.15	25.85	1.91
Diseases of the circulatory system	6	22.49	4.65	49.52	14.69
Diseases of the eye and adnexa	6	20.13	3.06	78.02	29.04
Diseases of the nervous system	7	37.03	24.32	67.30	15.26
Diseases of the respiratory system	6	16.90	2.01	25.74	11.60
Diseases of the skin and subcutaneous tissue	1	42.75	42.75	42.75	–
Endocrine, nutritional and metabolic diseases	13	24.72 *	12.78	48.60	13.86
Mental and behavioral disorders	2	25.68	17.36	34.00	11.77
Neoplasms	19	20.43	0.00	53.71	13.92
Other	4	25.10 *	21.65	28.55	3.98
Overall	92	24.25 *	0.00	78.02	14.18
Size of target population (upper level)	92	170203	15	3253000	499215

*Abbreviations*: *Min* minimum, *Max* maximum, *SD* standard deviation

All discounts presented in %

Assessment with “Major Benefit” = 13.64 % (TP upper level = 2333.33)

Assessment with “Less Benefit” = 9.00 % (TP upper level = 1460)

* *p* < 0.05 compared to mean discount of the other indications

Manufacturers’ own rating of the additional benefit was different from the rating of the additional benefit by IQWiG and/or GBA in 95/193 assessments and often overestimated. An identical rating between IQWiG and GBA (*n* = 95) led to an average discount of 22.14 % [0–78.02; SD 14.39] compared to a discount of 23.81 % [0–58.73; SD 13.79] in assessments without identical rating (*n* = 69). When renouncing a dossier submission (*n* = 5) the negotiated average discount was 31.51 % [3.97–78.02; SD 29.95] vs. 23.84 % [0–67.30; SD 13.0] with dossier submission and the GBA granted “no additional benefit” (*n* = 86).

Number of target populations assessed were more than one in 47/96 assessments. The overall size, measured by the upper level mentioned in the GBA decision, was *n* = 122,808 [6–3,253,000; SD 409,065]. For discounts by size of target population see Table 3.

**Table 3:** Target population and discount

Overall					
Size of target population	*n*	Mean	Min	Max	SD
0–< 1000	34	20.84	1.00	53.71	12.15
1000–< 2500	38	21.28	0.00	42.54	11.53
2500–< 7500	31	19.79	4.74	39.44	9.00
7500–< 25000	32	21.61	2.01	46.44	11.43
25000–< 150000	34	23.88	3.16	67.30	15.12
150000+	24	25.18	2.01	78.02	19.50
Orphan drugs					
Size of target population	*n*	Mean	Min	Max	SD
0–< 300	10	13.70	1.00	24.50	8.15
300–< 1500	10	20.66	9.00	29.78	6.52
1500+	9	20.81	10.96	25.54	4.66

All discounts presented in %

*Abbreviations*: *Min* minimum, *Max* maximum, *SD* standard deviation

#### Orphan drug status

Mean discount for orphan drugs (29/193) was 18.31 % [1.00–29.78; SD 7.27] compared to non-orphan drugs (164/193) 22.60 % [0–78.02; SD 13.83] (*p* = 0.015). When comparing orphan drugs only with non-orphans with additional benefit (71/193) 20.67 % [0–58.73; SD 13.21] the difference was not significant (*p* = 0.256). Table 4 presents the results per indication class and benefit category.

**Table 4:** Discounts by benefit categories and indication class (Orphan drugs)

Additional benefit: overall					
Indication class by ICD-10	*n*	Mean	Min	Max	SD
Diseases of the respiratory system	1	10.96	10.96	10.96	–
Diseases of the circulatory system	3	22.31	20.72	25.49	2.75
Neoplasms	16	19.59	9.00	29.78	5.82
Other	1	24.50	24.50	24.50	–
Endocrine, nutritional and metabolic diseases	6	12.43	1.00	25.31	10.17
Diseases of the digestive system	2	20.21	14.87	25.54	7.54
Overall	29	18.31	1.00	29.78	7.27
Size of target population (upper level)	29	1896	11	7850	2583
Additional benefit: considerable					
Indication class by ICD-10	*n*	Mean	Min	Max	SD
Neoplasms	2	20.80	19.78	21.81	1.44
Endocrine, nutritional and metabolic diseases	1	5.05	5.05	5.05	–
Overall	3	15.55	5.05	21.81	9.15
Size of target population (upper level)	3	2348	143	5000	2459
Additional benefit: minor					
Indication class by ICD-10	*n*	Mean	Min	Max	SD
Diseases of the circulatory system	3	22.31	20.72	25.49	2.75
Neoplasms	3	21.55	13.85	25.81	6.68
Other	1	24.50	24.50	24.50	–
Endocrine, nutritional and metabolic diseases	5	13.91	1.00	25.31	10.62
Diseases of the digestive system	1	25.54	25.54	25.54	–
Overall	13	19.32	1.00	25.54	8.21
Size of target population (upper level)	13	2167	11	7850	2924
Additional benefit: non-quantifiable					
Indication class by ICD-10	*n*	Mean	Min	Max	SD
Diseases of the respiratory system	1	10.96	10.96	10.96	–
Neoplasms	11	18.84	9.00	29.78	6.29
Diseases of the digestive system	1	14.87	14.87	14.87	–
Overall	13	17.93	9.00	29.78	6.21
Size of target population (upper level)	13	1521	25	7500	2393

All discounts presented in %

*Abbreviations*: *Min* minimum, *Max* maximum, *SD* standard deviation

#### Appropriate comparative therapy

For orphan drugs (*n* = 29) the additional benefit is preset by law independent from the chosen ACT. Table 5 shows that for 67/164 assessments the manufacturer did not follow the GBA recommendations regarding the ACT. For 83/164 assessments, more than one ACT was recommended by the GBA (30 of those not following the GBA and 53 who followed the GBA recommendation). When comparing the annual cost of the new drug to the cheapest ACT recommended by the GBA, annual costs were at least 54 % higher for those with more than one ACT.

**Table 5:** Acceptance of ACT and discounts (*N* = 164, excl. orphan drugs)

Acceptance of ACT set by GBA: no										
Additional benefit category	ACT ≤ 1					ACT > 1				
	*n*	Mean	Min	Max	SD	*n*	Mean	Min	Max	SD
Overall	37	20.25	3.40	67.30	13.21	30	22.46	0.00	53.71	11.88
Overall with additional benefit	18	16.63	4.74	39.65	10.05	9	19.72	0.00	51.67	15.30
Considerable	7	18.05	10.07	32.87	7.93	4	15.06	10.07	23.15	6.29
Minor	8	16.70	4.74	39.65	13.26	5	23.45	0.00	51.67	19.99
Non-quantifiable	3	13.16	10.07	19.35	5.36	0	–	–	–	–
No	19	20.25	3.40	67.30	13.21	21	23.63	3.16	53.71	10.30
Acceptance of ACT set by GBA: yes										
Additional benefit category	ACT ≤ 1					ACT > 1				
	*n*	Mean	Min	Max	SD	*n*	Mean	Min	Max	SD
Overall	44	24.14	0.00	78.02	15.22	53	23.07	2.01	58.73	14.25
Overall with additional benefit	17	23.26	6.35	35.19	12.60	27	22.05	2.01	58.73	14.69
Major	1	13.64	13.64	13.64	–	0	–	–	–	–
Considerable	7	19.05	6.35	35.19	11.70	8	29.08	10.38	42.54	14.21
Minor	7	31.58	19.80	46.44	11.77	15	18.78	2.01	58.73	16.10
Non-quantifiable	2	13.64	13.64	13.64	0.00	4	20.25	19.25	21.25	1.15
No	27	24.69	0.00	78.02	16.86	25	24.73	2.01	49.52	13.92
Additional harm potential	0	–	–	–	–	1	9.00	9.00	9.00	–

*Abbreviations*: *Min* minimum, *Max* maximum, *SD* standard deviation

All discounts presented in %

The mean discount did not significantly differ compared to those where the manufacturer followed the recommendation and no noticeable differences were seen regarding indication groups (Table 5).

For the assessments where the manufacturer deviated from GBA recommendation (*n* = 30), the manufacturer chose the more costly ACT in 19 assessments. Annual cost of the new compound ranged from 94–8100 % compared to ACT. Mean discount was 21.72 % [10.75–43.08; SD 6.56). In 11 assessments, the definition of subgroups was different so that no comparison was possible.

#### Incorporated HRQoL-data

In 49/193 assessments, the GBA incorporated HRQol-data in their assessment and granted an additional benefit for 33 (21 of them with minor). Overall, the mean discount for drugs with incorporated HRQoL-data was 19.29 % [0–40.77; SD 10.93] compared to those without incorporated HRQoL-data 22.87 % [0–78.02; SD 13.73], *p* = 0.100. No significant differences were shown for discounts per benefit category, indication class or drug status (orphan/non-orphan) (Table 6).

**Table 6:** Incorporated HRQoL data and discounts

Additional benefit category	Incorporated HRQoL-data									
	No					Yes				
	*n*	Mean	Min	Max	SD	*n*	Mean	Min	Max	SD
Overall	144	22.87	0.00	78.02	13.73	49	19.29	0.00	40.77	10.93
Overall with additional benefit	67	20.89	4.65	58.73	12.38	33	18.15	0.00	39.65	10.49
Major	1	13.64	13.64	13.64	–	0	–	–	–	–
Considerable	19	23.02	9.99	42.54	12.00	10	16.19	5.05	39.44	9.64
Minor	27	22.73	4.65	58.73	15.70	21	18.63	0.00	39.65	11.33
Non-quantifiable	20	16.74	9.00	29.78	5.33	2	22.99	19.04	26.93	5.58
No	76	24.80	0.00	78.02	14.65	16	21.66	2.01	40.77	11.77
Additional harm potential	1	9.00	9.00	9.00	–	0	–	–	–	–

*Abbreviations*: *HRQoL* health-related quality of life, *Min* minimum, *Max* maximum, *SD* standard deviation

All discounts presented in %

### Linear regression

The univariate regression provided significant results for factor “Rating” and “Indication”, which significantly correlate with the dependent variable (Appendix 1). For the multivariate regression, we included all significant variables. To avoid a non-inclusion of categories due to the low number of observations, we included all benefit categories and indication classes too. Running the multivariate regression, two of the included variables were excluded automatically and the variable with the maximum mean discount (“Diseases of the nervous system”, *n* = 9) was significant (Appendix 2). There were statistically significant differences between group means (*p* = 0.020). The model explained 16.3 % of the variations.

## Discussion

The present study analyses possible factors influencing the final negotiated discount after AMNOG-assessment. Therefore, results of this analysis should be interpreted as a trend and starting point for further research.

Of the analyzed 193 assessments, 91 rated with “no additional benefit”. This is nearly half of all assessments, whereas for orphan drugs (29/193) the law presets an additional benefit. As expected, a proven additional benefit influences the final discount positively. However, the rating itself seems not to fully “reflect” the expected discount. For example, 0 % given for a rating with minor additional benefit and 78.02 % for a rating with no additional benefit. Discounted prices negotiated based on the early benefit assessment show that the new system has resulted in substantial rebates within a wide range of results. However, free pricing for the first year seems not to impose a structurally negative impact [5].

The number of target populations assessed did not show any significant differences on final discount. For size of target population, there was only a hint that a smaller target population leads to lower discounts. This might reflect the circumstance that discounts for orphan drugs were lower, compared to non-orphan drugs.

Orphan drugs need to be treated differently due to the circumstance that the law presets their additional benefit. When separating orphan drugs, mean discount for orphan drugs was not significantly different to those with additional benefit and non-orphan drug status.

If the manufacturer does not deviate from GBA recommendations on the ACT, discounts seem to be smaller. When deviating from recommendation, assessments were granted with “no additional benefit” if no adequate reason existed. For half of all assessments, the GBA recommended more than one ACT. If the manufacturer chosed the more costly ACT, it had no significant impact on discounts.

The acceptance and consideration of HRQoL-data within the GBA appraisal tends to influence the final discount positively. In the future, measured quality of life data and other patient-reported outcomes might play a greater role in early benefit assessment [6]. For example, crizotinib was not able to show an improvement of overall survival and the GBA did not analyze the surrogate parameter progression-free survival to assess the benefit of the drug. However, the significant reduction in non-fatal symptoms and a significant improvement in quality of life led to a considerable additional benefit ruling in comparison to best supportive care [7].

Based on current evidence and chosen influence factors, it was not possible to develop a model that reliably explains how much the discount level is expected to change in response. With *R*^2^ = 0.163 the regression model explained only a small proportion of total variation [8]. On the one hand, this might be a result of the limited sample size and inter-correlation between independent variables. On the other hand, other influence factors and/or analysis tools might be necessary to consider (e.g. respective benchmarks introduced in the framework agreement signed by the GKV-SV and the manufacturers’ unions [9]). This seems the fact especially when predicting human behavior because the decision making process is overly complex: clarifying objectives, generating alternatives, define the BATNA (the “Best Alternative To Negotiated Agreement”), evaluating consequences and making tradeoffs. Some influencing factors may not be directly observable, such as the negotiation skills of the manufacturer, acceptance and “attitude” of the manufacturer, or “good will” in situations of high-unmet clinical need, to name just a few. Decisions must be made in the presence of multiple usually conflicting and incommensurate criteria or attributes, what makes a multi-criteria decision making approach reasonable [10].

For discount calculation, we used data from the official German drug price list “Lauertaxe” for the largest package (N3). Often smaller packages exist or the manufacturer decides to withdraw package sizes from the market. In addition to the negotiated discount, a manufacturer might lower the initial price that was set at market launch at their discretion any time before or after finalization of negotiations [11]. Regarding the prices and discounts it is important to note, that if one product covers different therapeutic areas with different ratings, mixed prices and/or discounts exist.

When evaluating the discount by assessment level (*n* = 96) the overall mean discount was 23.16 % [0–78.02 %; SD 14.66]. Hence, 92 subgroups assessed with “no additional benefit” within 96 assessments totally changes the picture. When taken “no additional benefit” as the highest rating granted, the number of assessments is 29 [2, 12, 13].

The small number of observations (especially when differing by benefit categories, drug status or indication) and the wide spread of negotiated discounts limit our analysis. Due to the limited group size, all significant results need to be interpreted with caution.

While the early benefit assessment and appraisal of clinical evidence itself is a very clear and transparent procedure, no official algorithm exists for a prediction of levels of negotiated price discounts [4, 14]. This is a result of confidentiality between the negotiation parties. Thus, a high uncertainty remains due to modalities regarding setting the maximum reimbursable price (e.g. premium over the price of the ACT or discount on an existing market price of the new product) and the proceedings of the arbitration board when negotiations fail.

Some hard criteria for the discount negotiation are mandatory and pre-set:

- Result of the GBA-assessment
- Price as set by the manufacturer
- Annual costs of comparable therapies

That leads to the presumption that the negotiation by itself is a “black box” with uncertain outcome and perhaps contrary to the interests of the individuals insured in the GKV [15]. This seems to be a fact, although the GBA is obliged to make considerations of economic efficiency when including a new health care service into the service catalogue.

From an economic standpoint, there are two approaches to explain price building for pharmaceuticals and for the assessment of functionality as well as appropriateness of negotiation criteria. The first approach calculates the price of a new pharmaceutical based on research and development costs (plus X for innovation). The second approach measures the willingness-to-pay in dependence of patient preferences of the society (assessing the properties and advantages of a product). Pareto-optimum might be reached when full information about preferences and willingness-to-pay and low transaction costs exist [4]. In reality, this is only true in a very limited number of cases. Hidden information and hidden action between market players and transaction costs are not known.

Under highly specific assumptions, it is possible to internalize occurring external effects with bilateral negotiations and reach pareto-efficient negotiation results. However, manufacturers do not negotiate with patients directly. GKV-SV acts on behalf of the sickness funds and representative for patients (insured community). Physicians influence the distribution of new drugs when prescribing drugs and there is a need for a reserve capacity of health services that has to be offered by the government independent of the actual demand.

In addition, monetization of the additional benefit by the arbitration board would also require the measurement of patients’ willingness-to-pay. Preferences and the willingness-to-pay of subjects (patients) are not known [11, 16] and the calculation of the reimbursable price is not able to consider this within its individual price components: result of benefit rating, price of ACT and undefined price if additional benefit is proven. Other unconsidered issues are incentives for innovation or optimal patient access [17].

Most other European countries focus more on cost-effectiveness approaches. For instance, the British National Institute for Health and Care Excellence (NICE), besides evaluating the “clinical effectiveness” of a new technology, also performs cost-effectiveness assessments. Cost-effectiveness approaches are also established in several other European countries such as Sweden, the Netherlands, Austria, and Poland [18, 19]. Hence, an assessment of the relation of benefits of a new compound to costs seems necessary for the preparation of centralized price negotiations [11, 20, 21].

In the United States, no organization performs drug assessments similar to those in Europe, although the relevance of a comparative effectiveness research has increased with the introduction of the American Recovery and Reinvestment Act of 2009 [22].

Discounts in Germany are based on early benefit assessment. Evaluations of the society’s willingness-to-pay and the welfare maximizing price cap are not relevant [15]. Of course, there is a high incentive for choosing the negotiation solution before doing a health economic evaluation. From a regulatory perspective, currently a health economic assessment conducted by IQWiG may only start the earliest 15 months after launch and might take up to 3 years. During that time, the arbitration award builds the foundation for reimbursement. From a scientific as well as pragmatic point of view, that implies a loss of producer surplus and patient benefits. When a manufacturer is presenting a new drug with additional benefit, he does not need a cost-benefit-assessment. If the new drug has no additional benefit, a cost-benefit-assessment would not change anything as per current regulations. If a manufacturer decides to provide cost-effectiveness results with the dossier, the GBA will certainly ignore that based on formal grounds. The GBA stated for the Ticagrelor case, that any cost offset that lower incremental costs of Ticagrelor should be discussed in the negotiation with the GKV-SV or could be become relevant in a cost-benefit analysis that any party can ask for post arbitration [23, 24].

Manufacturers value this process in a different way. On the one hand, incentives for the development of innovative drugs could be lower due to (sometimes misleading) assessments. One year after launch, they are no longer able to achieve monopolistic prices for their innovative products. On the other hand, when negotiating individual discounts it is possible to focus on individual product properties so that the final discount prevents the abuse of the monopolistic position [25].

As a final point, the negotiation procedure ties up public resources and may lead to overwhelming bureaucracy costs because of delayed negotiations and the inability to find a price (when going to the arbitration court). This raises the question what the real amount of budget impact and cost savings is [26].

## Conclusions

Negotiations alone seem not to be the appropriate way to find a price optimum for new drugs. With the influence factors assessed in this study, the results are not properly predictable. Each negotiation process starts under new conditions. With respect to the negotiated discounts, there is a potential risk that the aim of a mere cost reduction becomes dominant with significant spillover effects worldwide.

Decision-analytic assessments might help to fill potential gaps when planning different price strategies. For that reason, cost-benefit-assessment might be an essential tool for negotiations. Cost-benefit-assessments conducted by the manufacturer and/or payer at the time of dossier submission may help to give a first impression about the adequacy of the true value for a new drug.

## Appendix 1

**Table 7:** Results of the univariate regression^a^ (*N* = 193)

Predictor (X)	*n*	Beta	SE	Coeff (β)	*p*-value	R^2^
Additional benefit	100	−0.041	0.019	−0.156	0.030*	0.024
Major	1	−0.084	0.132	−0.046	0.527	0.002
Considerable	29	−0.015	0.027	−0.042	0.564	0.002
Minor	48	−0.014	0.022	−0.045	0.532	0.002
Non-quantifiable	22	−0.052	0.030	−0.127	0.078	0.016
No additional benefit	92	0.044	0.019	0.167	0.020*	0.028
Less benefit (additional harm potential)	1	−0.130	0.132	−0.071	0.324	0.005
Orphan status	29	−0.043	0.026	−0.117	0.104	0.014
Indication per category						
Certain infectious and parasitic diseases	51	0.001	0.022	0.002	0.975	0.000
Diseases of the blood and blood-forming organs	2	0.026	0.094	0.020	0.784	0.000
Diseases of the circulatory system	14	0.035	0.036	0.070	0.332	0.005
Diseases of the digestive system	6	0.056	0.054	0.075	0.303	0.006
Diseases of the eye and adnexa	7	−0.026	0.051	−0.037	0.609	0.001
Diseases of the genitourinary system	2	−0.022	0.094	−0.017	0.816	0.000
Diseases of the musculoskeletal system and connective tissue	1	0.176	0.131	0.096	0.183	0.008
Diseases of the nervous system	9	0.167	0.043	0.268	0.000**	0.072
Diseases of the respiratory system	9	−0.095	0.044	−0.152	0.034*	0.023
Diseases of the skin and subcutaneous tissue	1	0.209	0.131	0.114	0.113	0.013
Endocrine, nutritional and metabolic diseases	24	−0.024	0.029	−0.060	0.403	0.004
Mental and behavioral disorders	2	0.038	0.094	0.029	0.689	0.001
Neoplasms	57	−0.028	0.021	−0.097	0.181	0.009
Other	8	−0.013	0.048	−0.020	0.787	0.000
Number of ACT >1	83	0.016	0.019	0.059	0.418	0.003
Incorporated HRQoL-data	49	−0.036	0.022	−0.119	0.100	0.014

*Abbreviations*: *HRQoL* health-related quality of life, *Beta* standardized coefficient, *SE* standard error, *Coeff* coefficient, *R* ^*2*^ coefficient of determination

** Significant at the 0.01 level

* Significant at the 0.05 level

^a^ Dependent Variable (Y): price change (discount)

## Appendix 2

**Table 8:** Summary of the multivariate regression

Model Summary^a^					
R	R^2^		R^2^ corr		SE
0.404^a^	0.163		0.076		0.1262671
ANOVA^a^					
	SS	df	MS	F	*p*-value
Regression	0.540	18	0.030	1.882	0.020^*^
Residual	2.774	174	0.016	–	–
Total	3.314	192	–	–	–
Coefficients^a^					
Predictors (X)		Beta	SE	Coeff (β)	p-value
(constant)		0.228	0.021		0.000
Major benefit		−0.102	0.135	−0.056	0.451
Considerable benefit		−0.033	0.029	−0.091	0.249
Minor benefit		−0.029	0.024	−0.094	0.228
Non-quantifiable benefit		–0.060	0.031	–0.147	0.055
Less benefit		–0.138	0.128	–0.076	0.282
Diseases of the digestive system		0.061	0.055	0.080	0.270
Diseases of the genitourinary system		–0.002	0.093	–0.001	0.984
Endocrine, nutritional and metabolic diseases		–0.017	0.032	–0.042	0.608
Other		0.010	0.051	0.016	0.839
Diseases of the nervous system		0.156	0.047	0.252	0.001**
Diseases of the musculoskeletal system and connective tissue		0.199	0.129	0.109	0.124
Certain infectious and parasitic diseases		0.013	0.025	0.043	0.604
Diseases of the circulatory system		0.043	0.038	0.085	0.264
Diseases of the skin and subcutaneous tissue		0.199	0.128	0.109	0.122
Diseases of the blood and blood-forming organs		0.017	0.092	0.013	0.856
Diseases of the eye and adnexa		–0.029	0.052	–0.041	0.575
Diseases of the respiratory system		–0.086	0.046	–0.138	0.064
Mental and behavioural disorders		0.028	0.092	0.022	0.757

Predictors “Neoplasms” and “No additional benefit” automatically excluded from the model

*Abbreviations*: *Beta* standardized coefficient; *df* degrees of freedom; *MS* mean squares (SS/dfs); *F* F statistic; *R* correlation coefficient; *R* ^*2*^ coefficient of determination; *R* ^*2*^ *corr* coefficient of determination corrected; *SE* standard error; *SS* sums of squares

** Significant at the 0.01 level

* Significant at the 0.05 level

^a^ Dependent Variable (Y): price change (discount)

## Abbreviations

ACT: appropriate comparative therapy
AMNOG: Arzneimittelmarkt-Neuordnungsgesetz
GBA: Gemeinsamer Bundesausschuss (Federal Joint Committee)
GKV: Gesetzliche Krankenversicherung (German Statutory Health Insurance)
GKV-SV: Spitzenverband Bund der Krankenkassen (National Association of Statutory Health Insurances Funds)
HRQoL: health-related quality of life
IQWiG: Institut für Qualität und Wirtschaftlichkeit im Gesundheitswesen
NICE: National Institute for Health and Care Excellence
